# LncRNA ANCR down-regulation promotes TGF-β-induced EMT and metastasis in breast cancer

**DOI:** 10.18632/oncotarget.18622

**Published:** 2017-06-27

**Authors:** Zhongwei Li, Meichen Dong, Dongmei Fan, Pingfu Hou, Hongyuan Li, Lingxia Liu, Cong Lin, Jiwei Liu, Liangping Su, Lan Wu, Xiaoxue Li, Baiqu Huang, Jun Lu, Yu Zhang

**Affiliations:** ^1^ The Key Laboratory of Molecular Epigenetics of Ministry of Education (MOE), Northeast Normal University, Changchun, China; ^2^ The Institute of Genetics and Cytology, Northeast Normal University, Changchun, China; ^3^ Jiangsu Center for the Collaboration and Innovation of Cancer Biotherapy, Cancer Institute, Xuzhou Medical University, Xuzhou, China

**Keywords:** lncRNA ANCR, TGF-β, EMT, RUNX2, metastasis

## Abstract

Epithelial to mesenchymal transition (EMT) is a progression of cellular plasticity critical for development, differentiation, cancer cells migration and tumor metastasis. As a well-studied factor, TGF-β participates in EMT and involves in physiological and pathological functions of tumor progression. Accumulating evidence indicates that long noncoding RNAs(lncRNAs) play crucial roles in EMT and tumor metastasis. Here, we find that lncRNA ANCR participates in TGF-β1-induced EMT. By our ChIP and Real-time PCR assays, we reveal that TGF-β1 down-regulates ANCR expression by increasing HDAC3 enrichment at ANCR promoter region, which decreases both H3 and H4 acetylation of ANCR promoter. In addition, by western blot and transwell assays, we indicate that ectopic expression of ANCR partly attenuates the TGF-β1-induced EMT. Downstream, ANCR inhibits breast cancer cell migration and breast cancer metastasis by decreasing RUNX2 expression *in vitro* and *in vivo*. Thus, our study identifies ANCR, as a new TGF-β downstream molecular, is essential for TGF-β1-induced EMT by decreasing RUNX2 expression. These results implicate that ANCR might become a prognostic biomarker and an anti-metastasis therapy target for breast cancer.

## INTRODUCTION

Breast cancer is the most common cancer in women worldwide [1, 2], and the distance metastasis is the major cause for the breast cancer mortality [3, 4]. The epithelial-mesenchymal transition (EMT) program was first found in embryonic development. EMT program induces cell-cell conjunctions decreasing and down-regulation of epithelial marker (E-cadherin), while it promotes cell motility and increases the expression of mesenchymal markers (N-cadherin, Vimentin, Fibronectin). EMT program has also been proven to play a core part in breast cancer cell migration and metastasis in breast cancer patients [5, 6].

The transforming growth factor-β (TGF-β) is a classical inducer of EMT, and also a key factor for EMT maintenance in a variety of epithelial cells or epithelial-like cancer cells in culture; while it also contributes to tumor metastasis *in vivo* [7–9]. Studies have indicated that cancer cells promote the production of active TGF-β and TGF-β receptor, which facilitates or is necessary for the induction of EMT in cancer cells [10]. Moreover, the TGF-β-mediated activation of Smad-cascade has also been revealed playing an important role in EMT associated tumor progression [8, 9]. Meanwhile, some EMT key regulatory factors, such as Zeb1, Twist, Snail and Slug, which participate in cancer progression or cancer metastasis, are also involved in TGF-β1-induced EMT [11–13]. Our previous study also discovered that SOX4, a member of the C subgroup of SOX (SRY-related HMG box) transcription factor family, and CDK5 (Cyclin-dependent kinase 5), were up-regulated in TGF-β1-induced EMT [14, 15].

Long noncoding RNAs (lncRNAs) are noncoding RNAs of over 200 nucleotides. LncRNAs have many effects on human diseases, cell proliferation, differentiation and cancer cells migration [16–19]. Studies have shown that some lncRNAs play central roles in tumor metastasis [16]. Noticeably, some lncRNAs have been proven involved in TGF-β1-induced EMT [20], including MALAT1, lnc-ATB, linc-RoR and lncRNA-Smad7 [18, 21–23].

ANCR (anti-differentiation noncoding RNA, ANCR) is an 855-nucleotide lncRNA, which is down-regulated during differentiation. ANCR is indispensable to enforce the undifferentiated cell state within epidermis [17]. Our previous study has demonstrated that ANCR interacts with EZH2 and promotes the CDK1-EZH2 binding to increase its phosphorylation at Thr-345 and Thr-487 residues, resulting in EZH2 ubiquitination and degradation in breast cancer cells [24]. This study aimed to further elucidation of the physiological and pathological functions of ANCR in breast cancer metastasis. The TGF-β-induced EMT is an essential cellular differentiation process that affects tissues as a coordinated unit in the embryogenesis and organogenesis [25], whereas ANCR inhibits cell differentiation [17]. In view of the opposing effects of TGF-β and ANCR in differentiation, we wonder if ANCR participates in TGF-β signaling pathway that mediates EMT and breast cancer metastasis.

Runt-related transcription factor 2 (RUNX2) is a member of polyomavirus enhancer-binding protein 2/core-binding factor superfamily. RUNX2 is known for its contribution to osteoblast phenotype and bone formation. In recent years, increasing attention has been focused on the relationship of RUNX2 and tumorigenesis [26–31]. Expression and function of RUNX2 have been implicated in various human cancers, especially in breast cancer. RUNX2 and its target genes are highly expressed in breast cancer tissues and play pivotal roles in breast cancer bone metastasis [30, 32–36]. For example, estradiol antagonizes the pro-metastatic activity of RUNX2 *in vitro*, and inhibits RUNX2-induced EMT and invasiveness of breast cancer cells [37]. In addition, high RUNX2 expression is significantly correlated with ER negative breast cancer [38]. RUNX2 also participates in TGF-β signal pathway and in TGF-β-induced EMT, as RUNX2 is upregulated in TGF-β1-treated thyroid carcinomas cells [29]. However, the mechanisms of the function of RUNX2 increase in TGF-β1-induced EMT have not been well studied. Besides, it has been reported that ANCR can repress RUNX2 expression during osteoblast differentiation in hFOB1.19 cell [19].

Based on exist reports as well as our previous study, we speculate that ANCR may play a role in TGF-β signal pathway and in TGF-β1-induced EMT. As concern mechanism, we explored the epigenetic modification of ANCR promoter and found the acetylation modification decreased by TGF-β1. Furthermore, we found that ANCR participated in TGF-β signal pathway by decreasing RUNX2 expression. In addition, we uncovered a negatively correlated expression pattern between ANCR and RUNX2 in breast cancer tissues and several breast cancer cell lines. Overall, data from our study identify a novel mechanism of ANCR function by participating in EMT and metastasis, and provide a new clue for the diagnosis and treatment of breast cancer.

## RESULTS

### ANCR was repressed in TGF-β1-induced EMT

**Figure 1:**
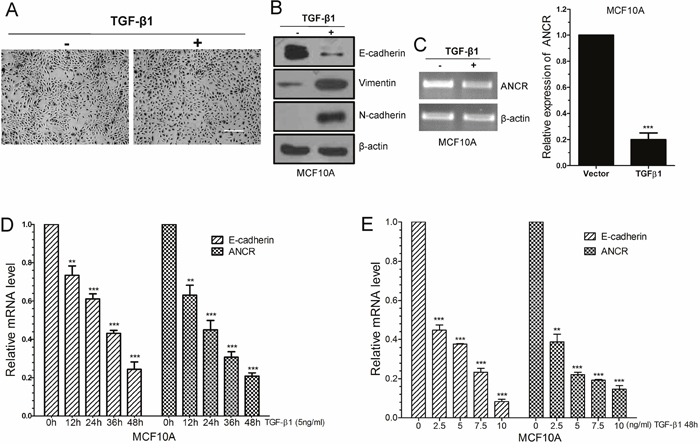
LncRNA ANCR was down-regulated in TGF-β1 induced EMT **(A)** The morphological change of MCF10A cells after treated by TGF-β1, as examined by phase contrast microscopy. Scale bars: 200 μm. **(B)** Western blots of the epithelial marker E-cadherin, and the mesenchymal markers (N-cadherin, Vimentin) in MCF10A treated by TGF-β1. **(C)** RT-PCR and Real-time PCR analysis of ANCR expression in TGF-β1treated MCF10A cells. **(D)** Real-time PCR analysis of ANCR and E-cadherin mRNA expression upon TGF-β1 treatment in different time. **(E)** Real-time PCR analysis of ANCR and E-cadherin mRNA expression upon TGF-β1 treatment in different dose. The data are presented as the mean±S.D. (n =3; **P<0.01, ***P<0.001, Student’s t-test).

TGF-β has been reported as a potent inducer and a key maintenance factor of EMT *in vivo* and *in vitro* [7]. Firstly, to determine whether ANCR participates in TGF-β1-induced EMT, TGF-β1 (10 ng/ml, 24h) was used to induce EMT program in immortalized non-transformed human epithelial cell line MCF10A. Under this model, normal MCF10A cells maintained their cobblestone-like morphology with tight cell-cell contact, whereas cells cultured with TGF-β1 exhibited an elongated fibroblast-like morphology with scattered distribution (Figure 1A). We then examined both the epithelial and mesenchymal markers by using western blotting. As can be seen, MCF10A cells cultured with TGF-β1 exhibited a dramatic downregulation of epithelial marker E-cadherin; with a concurrent upregulation of the mesenchymal markers N-cadherin and Vimentin (Figure 1B).

We tested and compared ANCR expression in TGF-β1-induced EMT model with that in normal MCF10A cells, and we observed a dramatic downregulation of ANCR in TGF-β1-induced EMT cells, as revealed by RT-PCR and real-time-PCR analysis (Figure 1C). We further investigate the relevance of ANCR with TGF-β1, and we found that ANCR expression responded to TGF-β1 in concentration- and time-dependent manners. As we can see, down-regulation of E-cadherin mRNA level was related to TGF-β1 treatment in a concentration-dependent and a time-dependent manner (Figure 1D and 1E). Meanwhile, the expression of ANCR was also much less with stronger TGF-β1 treatment (Figure 1D and 1E). Besides, we also indicated that ANCR was decreased in MCF7 cells when treated by TGF-β1(Supplementary Figure 1).

Together, these results demonstrate that ANCR is down-regulated during the TGF-β1-induced EMT in MCF10A cells.

### Ectopic expression of ANCR suppressed TGF-β1-induced EMT

**Figure 2:**
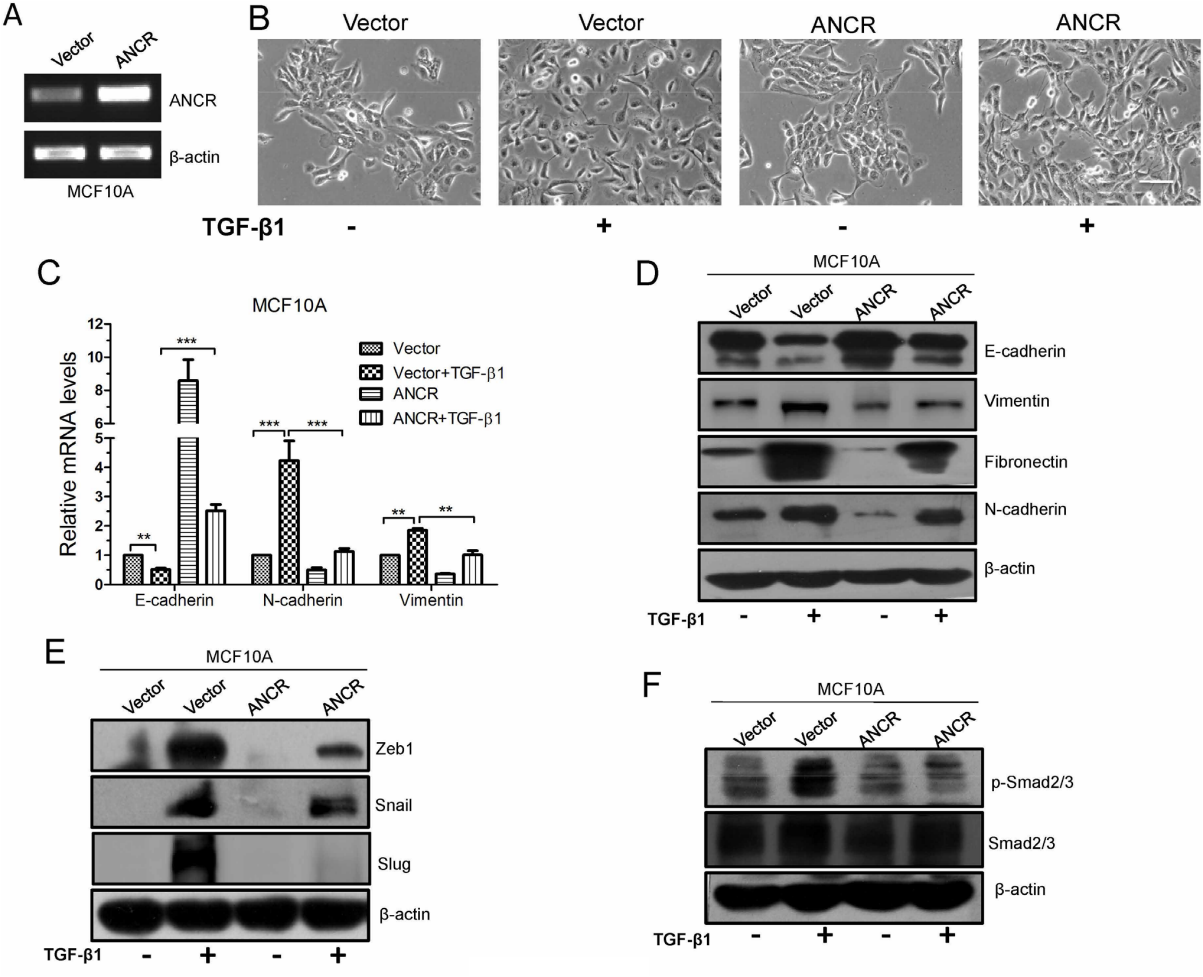
Overexpression of ANCR suppressed TGF-β1 induced EMT **(A)** ANCR overexpression was confirmed by RT-PCR after lentivirus infection in MCF10A cells. **(B)** The morphological change of MCF10A-Vector and MCF10A-ANCR cells after treated by TGF-β1, Scale bars: 100 μm. **(C)** mRNA expression of EMT markers E-cadherin, N-cadherin and Vimentin were assessed by real-time PCR in MCF10A cells cultured with or without TGF-β1 after infection of ANCR or empty vector. The data are presented as the mean±S.D. (n =3; **P<0.01, ***P<0.001, Student's t-test). **(D)** Immunoblotting analysis of expression EMT markers E-cadherin, Vimentin, Fibronectin and N-cadherin in MCF10A cells cultured without or with TGF-β1 after infection of ANCR or empty vector. **(E-F)** Western blot analysis of Zeb1, Snail, Slug and p-Smad2/3, Smad2/3 proteins expression in MCF10A-Vector and MCF10A-ANCR cells without or with TGF-β1.

The above data that ANCR was decreased during TGF-β1-induced EMT implicatd a possible role of ANCR in repression of TGF-β1-induced EMT process. To further validate the functional role of ANCR, we ectopically expressed ANCR in MCF10A cells using ANCR lentiviral infection, and the overexpression efficiency of ANCR was confirmed by RT-PCR (Figure 2A). Then we added TGF-β1 into the culture medium of MCF10A cells that were stably integrated with control Vector or ANCR. We observed that the MCF10A-Vector cells treated with TGF-β1 displayed an elongated fibroblast-like morphology with scattered distribution, whereas the MCF10A-ANCR cells treated with TGF-β1 almost retained a cobblestone-shaped epithelial morphology (Figure 2B). Further, we examined both epithelial and mesenchymal markers, and the results showed that treatment of MCF10A-ANCR cells with TGF-β1 increased expression of epithelial marker E-cadherin, while it decreased expression of mesenchymal markers N-cadherin, Vimentin and Fibronectin, in comparison to that in MCF10A-Vector cells (Figure 2C and 2D). Thus, our gain-of-function study pointed to a critical role of ANCR in TGF-β1-induced EMT.

In addition, we also detected the expression of EMT-related transcription factors, including Snail, Slug and Zeb1 in MCF10A-Vector and MCF10A-ANCR cells after treated with TGF-β1. The result demonstrated that ectopic expression of ANCR attenuated the TGF-β1-induced up-regulation of Snail, Slug and Zeb1 (Figure 2E). These findings suggest the fact that ANCR plays a critical role in TGF-β1-induced EMT in MCF10A cells.

Besides, we evaluated the effect of ANCR expression on TGF-β signaling pathway. To do so, the phosphorylation of Smad2/3, a well-known molecule involved in TGF-β1, was detected by western blotting. We found that overexpression of ANCR dramatically inhibited TGF-β1-induced phosphorylation of Smad2/3 (Figure 2F). These data indicate that ANCR can attenuate TGF-β signal pathway, at least partially, through inhibiting Smad2/3 phosphorylation.

Together, these data indicate that ANCR is able to partly reverse the TGF-β-induced EMT.

### Overexpression of ANCR inhibited TGF-β1-induced invasion and migration of MCF10A cells

**Figure 3:**
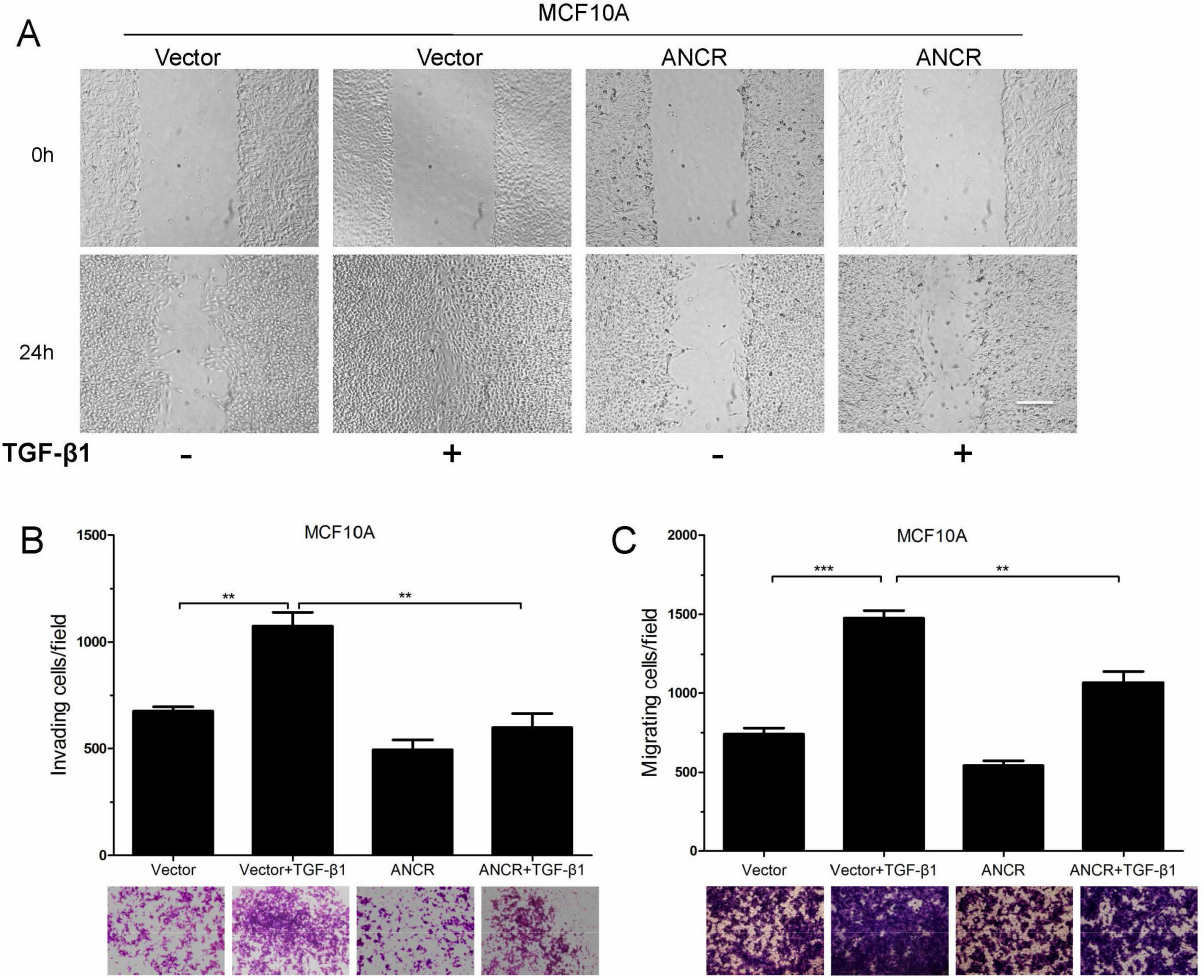
Overexpression of ANCR inhibited TGF-β1 induced migration **(A)** Representative images from wound healing assay in MCF10A-Vector and MCF10A-ANCR with TGF-β1. Scale bars: 200 μm. **(B-C)** Invasion and migration assays in MCF10A-Vector and MCF10A-ANCR cells after treated with or without TGF-β1. (n=3;* P<0.05,** P<0.01,*** P<0.001).

As widely accepted, TGF-β1-induced EMT has a great effect on cell migration and invasion. We then further investigated the function of ANCR in TGF-β1-induced cell motility. Our results showed that overexpression of ANCR decreased TGF-β1-mediated wound closure ability in MCF10A cells (Figure 3A). ANCR also decreased the migration and invasion ability induced by TGF-β1 in MCF10A cells (Figure 3B and 3C). Collectively, these data show that ANCR is able to repress TGF-β1-induced cells migration and invasion.

### TGF-β1 decreased ANCR expression by regulating the histone acetylation at ANCR promoter

**Figure 4:**
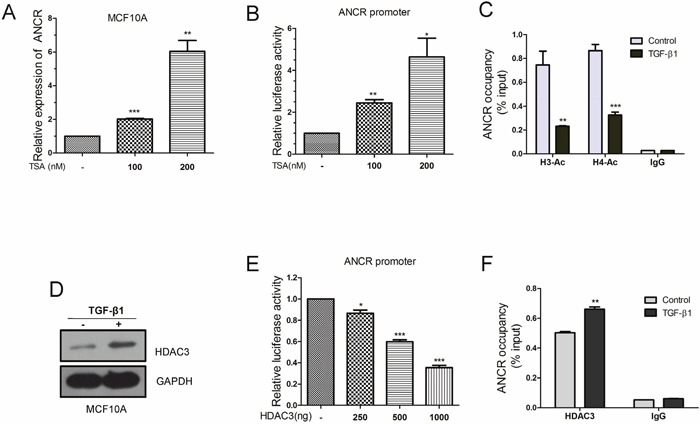
ANCR promoter region acetylation was regulated by TGF-β1 **(A)** Real-time PCR analysis of ANCR expression upon TSA treatment under different dose. **(B)** Luciferase reporter assays analysis of ANCR promoter activity by TSA. **(C)** ChIP-qPCR analysis of histone H3-acetylation and H4-acetylation enrichments on ANCR promoter region when treated with TGF-β1. **(D)** Western blot analysis of HDAC3 protein when MCF10A cells treated with TGF-β1. **(E)** Luciferase reporter assays analysis of ANCR promoter activity by overexpression of HDAC3. **(F)** DNA binding ability of HDAC3 on ANCR promoter was measured by ChIP-qPCR using anti-HDAC3 antibody or IgG antibody when treated with or without TGF-β1. The data are presented as the mean±S.D. (n =3; *P<0.05, **P<0.01, ***P<0.001).

Next, we wanted to figure out the mechanism how ANCR was inhibited in TGF-β-associated EMT and migration. A great deal of studies has demonstrated that histone acetylation is involved in gene expression regulation and in EMT program mediated by TGF-β [39–41]. For instance, TSA (trichostatin A), a histone deacetylation inhibitor, suppresses TGF-β1-induced EMT in hepatocyte cells by epigenetic modulation of type I collagen [41]. Histone deacetylases (HDACs), such as HDAC1 and HDAC3, have also been reported to repress TGF-β-induced EMT by increasing E-cadherin expression [40]. We were curious to find out if TGF-β attenuates ANCR transcription through modulating histone acetylation status of ANCR promoter. As the results, we found that ANCR expression was increased in MCF10A and MCF7 cells when treated with TSA (Figure 4A and Supplementary Figure 2). These results suggest that ANCR might be regulated by acetylation modification of histones. Indeed, TSA was able to increase ANCR promoter activity (Figure 4B), and ChIP experiments also indicated that the acetylation of histone H3 and H4 were reduced at the ANCR promoter region when treated with TGF-β1 (Figure 4C), suggesting that TGF-β1 represses ANCR expression probably by regulating histone acetylation of ANCR promoter.

Furthermore, we tested the expression of several HDAC family members (HDAC1, HDAC2, HDAC3 and HDAC4) in TGF-β-induced EMT model (Figure 4D and Supplementary Figure 3). Interestingly, we only discovered that HDAC3 was strongly elevated when treated with TGF-β1 in MCF10A cells (Figure 4D). Moreover, HDAC3 decreased ANCR promoter activity as revealed by luciferase reporter assays (Figure 4E). Besides, ChIP-qPCR data also revealed that the binding of HDAC3 on ANCR promoter region was increased in TGF-β1-treated MCF10A compared with control MCF10A cells (Figure 4F).

Together, our data demonstrate that TGF-β1 down-regulates ANCR expression through promoting HDAC3 enrichment at ANCR promoter region, which leads to the decrease of histone acetylation level at ANCR promoter, finally resulting in ANCR repression.

### ANCR attenuated TGF-β-induced EMT and migration by decreasing RUNX2 expression

**Figure 5:**
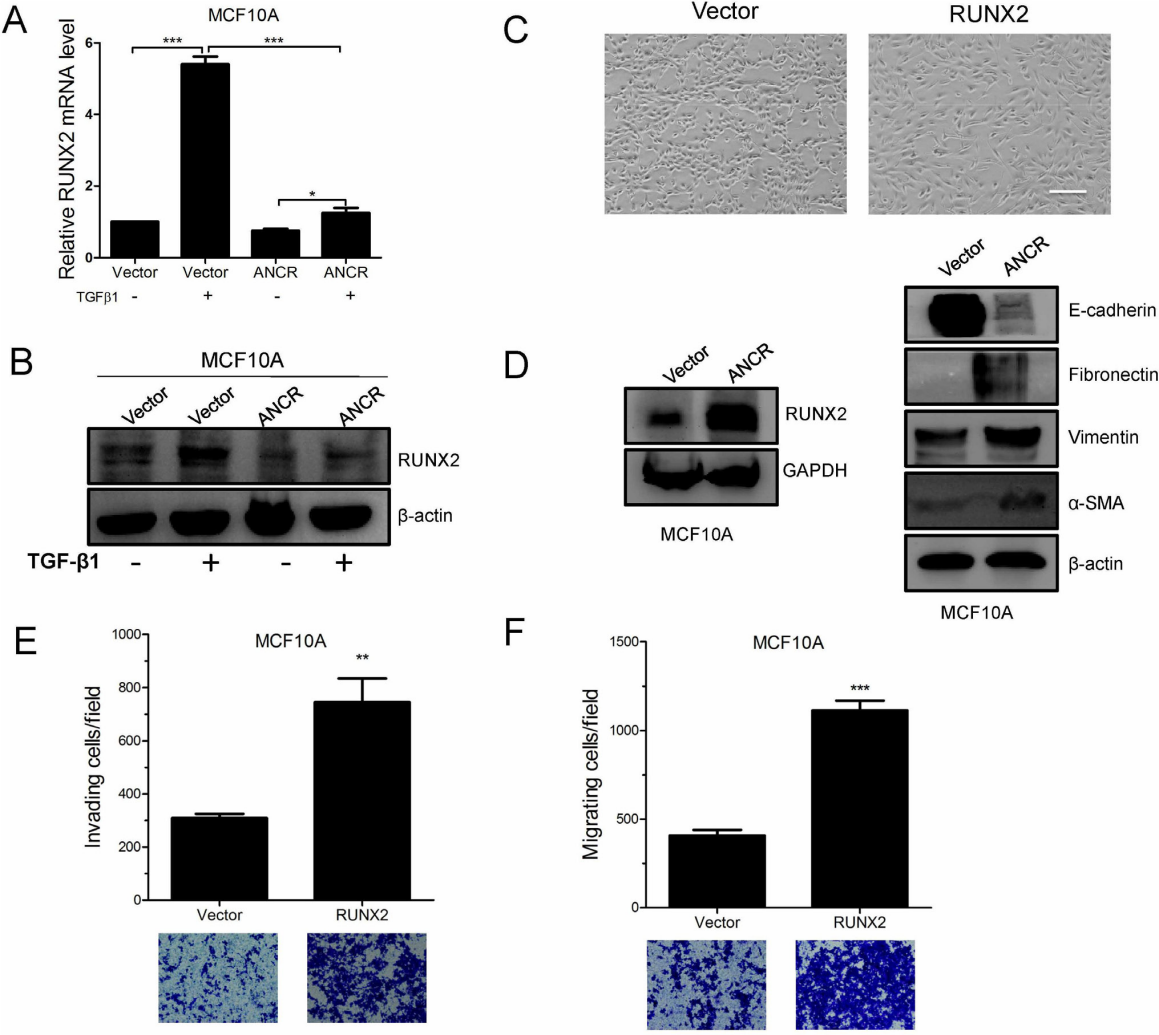
ANCR attenuated TGF-β1 induced EMT and migration by decreased RUNX2 expression **(A-B)** RUNX2 mRNA and protein level was assessed by Real-time PCR and western blot assays in MCF10A-Vector and MCF10A-ANCR cells. **(C)** The morphological change of MCF10A-RUNX2 cells, Scale bars: 200 μm. **(D)** Western blots of the epithelial marker E-cadherin, and the mesenchymal markers (Fibronectin, Vimentin, α-SMA) in MCF10A-Vector and MCF10A-RUNX2 cells. **(E-F)** Invasion and migration assays in MCF10A-Vector and MCF10A-RUNX2 cells. (n=3;* P<0.05,** P<0.01,*** P<0.001).

We next wanted to investigate the downstream mechanism by which ANCR attenuates the TGF-β1-induced EMT and migration. ANCR was first shown to maintain the undifferentiated state in somatic progenitor populations of epidermis [17], and this prompted us to proposed that ANCR might be able to regulate some key differentiation-associated transcription factors in TGF-β1-induced EMT model. It is well known that RUNX2 is a core transcription factor regulating many kinds of cell differentiation [19, 42, 43]. Besides, several studies have shown that RUNX2 is up-regulated in TGF-β1-induced EMT model [44, 45]. We speculated that ANCR participates in TGF-β1-induced EMT by regulating transcription factor RUNX2 expression. To test this hypothesis, we treated MCF10A-Vector and MCF10A-ANCR cells with TGF-β1 before assessing RUNX2 expression. Interestingly, RUNX2 was sharply increased at both mRNA and protein level upon treatment of TGF-β1 in MCF10A-Vector cells. In contrast, in MCF10A-ANCR cells, RUNX2 was only moderately increased by TGF-β1 (Figure 5A and 5B). Furthermore, we overexpressed RUNX2 in MCF10A cells, and confirmed RUNX2 overexpression by western blot (Figure 5D). We indicated that ectopic expression of RUNX2 induced EMT in MCF10A cells and RUNX2 promoted MCF10A cell migration and invasion (Figure 5C-5F).

**Figure 6:**
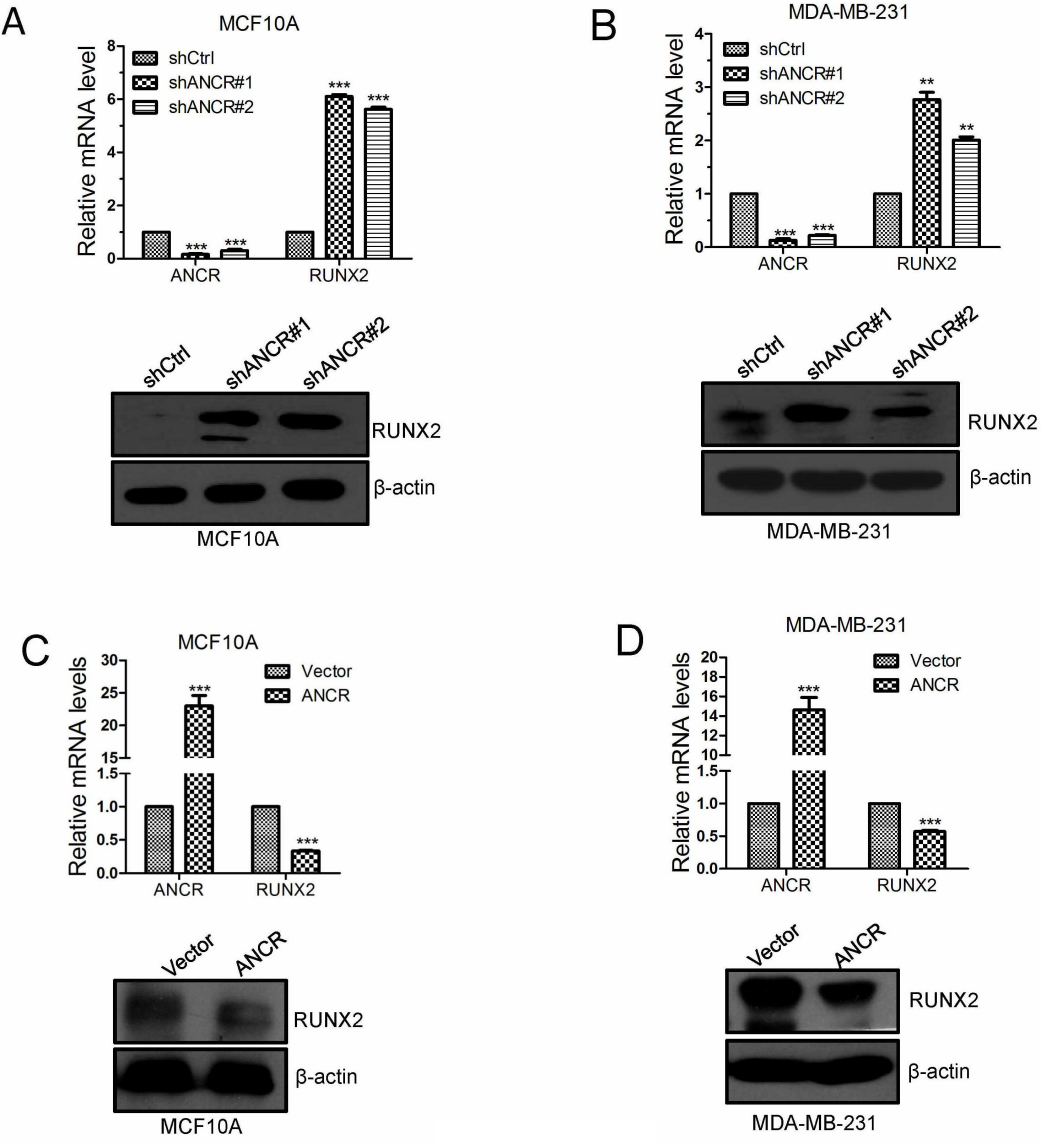
ANCR decreased RUNX2 expression in breast cancer cells **(A-B)** RUNX2 mRNA and protein expression was assessed by Real-time PCR and western blot when overexpression of ANCR in MCF10A and MDA-MB-231 cell lines. **(C-D)** RUNX2 mRNA and protein expression were assessed by Real-time PCR and western blot when knocking down ANCR in MCF10A and MDA-MB-231 cells. The data are presented as the mean±S.D. (n =3; **P<0.01, ***P<0.001, Student’s t-test).

Based on the previous data, we proposed that ANCR was able to down-regulation RUNX2 expression. Indeed, the expression of RUNX2 was up-regulated at both mRNA and protein level when ANCR was knocked down in MCF10A or MDA-MB-231 cells (Figure 6A and 6B); while RUNX2 was decreased at both transcriptional and protein level (Figure 6C and 6D). Besides, ectopic expression of ANCR also repressed RUNX2 expression in MCF7 cells and HEK293T cells (Supplementary Figure 4A-4D).

Based on these data, we propose that ANCR participates in TGF-β1-induced EMT through decreasing RUNX2 expression.

### ANCR decreased breast cancer metastasis *in vivo* by down-regulation of RUNX2

**Figure 7:**
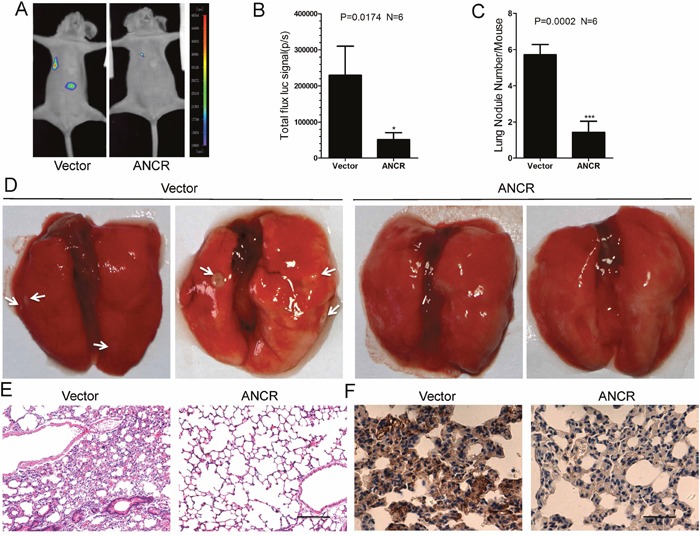
ANCR inhibited breast cancer metastasis *in vivo* by down regulation of RUNX2 **(A-B)** Representative bioluminescence images of lung metastasis in mice injected with cells as indicated via tail veins, and the metastasis were quantified by measuring the photo flux. (*P<0.5, Student’s t-test). **(C-E)** After 9 weeks, nude mice were sacrificed and lung metastatic nodules were examined macroscopically or detected by H&E staining. The white arrows denoted the metastatic nodules. Error bars in C represent mean± S.D. (***P<0.001, Student’s t-test). Scale bars: 100μm. **(F)** Representative images of the immunohistochemical staining of RUNX2 in nude mice lung metastasis sections. Scale bars: 50μm.

Furthermore, we intended to evaluate the pathological relevance between ANCR and tumor metastasis in breast cancer *in vivo*. The MDA-MB-231-Vector or MDA-MB-231-ANCR cells were injected into the tail veins of BABL/c nude mice. We found that MDA-MB-231-ANCR cells metastasized to the lungs of BABL/c nude mice less effectively, as illustrated by the bioluminescence imaging (Figure 7A and 7B). Noticeably, histological examination revealed that all nude mice bearing MDA-MB-231-Vector cells had a larger number of macroscopic lung metastases compared with nude mice transplanted with MDA-MB-231-ANCR cells (Figure 7C and 7D).

Subsequently, the lung tissue sections were prepared and examined after staining with hematoxylin and eosin, and we detected smaller and fewer metastatic foci in the tissues from nude mice injected with MDA-MB-231-ANCR cells (Figure 7E). Besides, we measured the RUNX2 level in lung tissue sections by the immunohisto-chemistry (IHC). We uncovered a reduced RUNX2 level in nude mice lung tissues injected with MDA-MB-231-ANCR cells (Figure 7F).

Taken together, these data suggests that ANCR is able to inhibit RUNX2 expression, which plays a pivotal role in repression of breast cancer metastasis *in vivo*.

### ANCR was probably negative correlated with RUNX2 expression in breast cancer tissues and breast cancer cell lines

**Figure 8:**
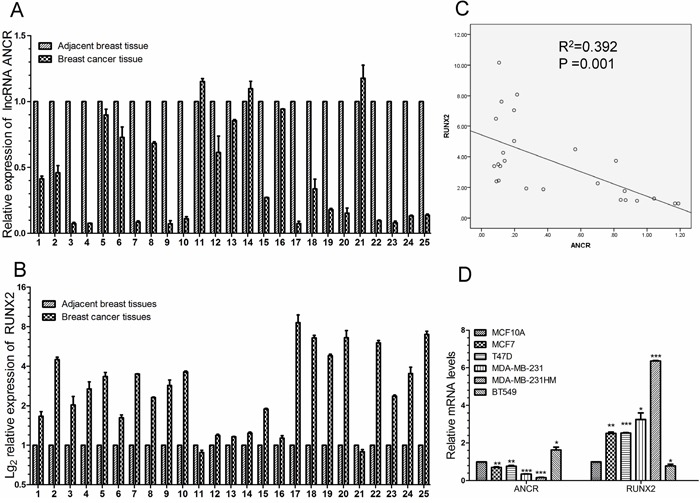
ANCR was negatively correlated with RUNX2 in human breast cancer tissues and breast cancer cell lines **(A-B)** Real-time PCR analysis of ANCR and RUNX2 expression in 25 human breast cancerous tissues and non-cancerous tissues. **(C)** Pearson correlation analysis of ANCR and RUNX2 in collected breast cancer tissues. **(D)** ANCR and RUNX2 expression level in breast cancer cell lines compared with in MCF10A. ANCR and RUNX2 mRNA level were normalized to β-actin. The data are presented as the mean± S.D. (n=3; *P<0.05, **P<0.01, ***P<0.001, Student’s t-test).

RUNX2 is known to be frequently up-regulated in breast cancer patients, and higher RUNX2 level often leads to the poor prognosis of patients [33, 36, 38]. Based on our finding that RUNX2 expression was modulated by ANCR, we speculated that the expression of ANCR might also be negatively correlated with RUNX2 in breast cancer patients and breast cancer cell lines. We then assessed the expression of ANCR and RUNX2 in a panel of paired tumor and normal primary tissue specimens collected from breast cancer patients (N=25). As can be seen, ANCR exhibited an apparently lower level in tumor tissues compared with the adjacent normal tissues (Figure 8A). In contrast, RUNX2 mRNA was much higher in tumor tissues (Figure 8B). Statistically, ANCR had a negative correlation with RUNX2 expression as judged by Pearson correlation analysis (Figure 8C). Our results demonstrated that the negative correlation between ANCR and RUNX2 expression may be universal in breast cancer patients. Finally, we detected the expression of ANCR and RUNX2 in breast cancer cell lines (MCF7, T47D, MDA-MB-231, MDA-MB-231HM and BT549), compared with human normal mammary epithelial cell MCF10A. The results revealed that ANCR expression may be negatively correlated with RUNX2 expression in breast cancer cell lines (Figure 8D).

Thus, our data suggest that ANCR and RUNX2 expression may have a close negative correlation. Also, we propose that low level of ANCR probably is associated with high level of RUNX2 in breast cancer tissues and breast cancer cell lines.

## DISCUSSION

Studies have revealed that some lncRNAs participate in TGF-β-induced EMT and TGF-β signal pathway. In this study, we determined ANCR as a novel downstream molecule of TGF-β signal pathway. ANCR was repressed by TGF-β, and it inhibited breast cancer cell migration and invasion. Furthermore, ANCR attenuated TGF-β1-induced EMT mainly by directly repressing RUNX2 expression. We also found that the expression of ANCR was lower in breast cancer tissues compared with the matched normal adjacent tissues, indicating that ANCR may act as a tumor suppressor. More interestingly, we found that the ANCR expression was negatively correlated with RUNX2 in breast cancer cell lines. Finally, we confirmed the negative association of low ANCR expression and high RUNX2 expression in breast cancer samples. Base on these data, we conclude that ANCR is an anti-metastatic lncRNA, and ANCR may be a useful prognostic biomarker to identify patients at a higher risk of breast cancer progression.

Acetylation is a core modification of histones in regulation of gene transcription. Our results demonstrated that TGF-β1 decreased ANCR promoter acetylation, and increased HDAC3 enrichment at the ANCR promoter (Figure 4C and 4F). Nevertheless, how the binding of HDAC3 increases the ANCR promoter activity remains to be explored. In general, some transcription factors are able to recruit HDACs, then to guide the HDACs to the target gene promoter. For instance, it has been reported that Snail can bind with HDAC1 and HDAC2 to repress E-cadherin transcription by decreasing the acetylation of the promoter region [39]. We speculate that some transcription factors might also be associated with HDAC3 and can bind on ANCR promoter region, leading to the increased recruitment of HDAC3 at ANCR promoter.

Our previous research has demonstrated that ANCR was able to repress EMT program through reduction of EZH2 stability. However, we and others have confirmed that the EZH2 mRNA and protein levels remain unchanged when treated with TGF-β1 [46]. This fact implicates that ANCR may participate in the TGF-β1-induced EMT through other molecular mechanisms. And we discovered that ANCR could inhibit RUNX2 expression to attenuate TGF-β1-induced EMT. Although our previous study has indicated that knockdown of ANCR is able to induce EMT by increasing EZH2 stability; in this study, our data uncover a new mechanism of ANCR participation in TGF-β1-induced EMT.

As a factor involved in differentiation, it has been reported that RUNX2 is increased in TGF-β signaling pathway. However, the mechanism of how TGF-β increases RUNX2 expression is unclear. Some studies suggest that TGF-β receptor promotes Smad phosphorylation and induces RUNX2 transcription [44]; however, solid evidence to confirm the direct regulation of RUNX2 expression is yet to be established. In this study, we showed that ectopic expression of ANCR was able to decrease RUNX2 expression both at mRNA and protein level, whereas knockdown of ANCR increased RUNX2 expression. Our results suggest that ANCR probably can directly regulate RUNX2 transcriptional expression, but the detailed insight of mechanism needs further study.

**Figure 9:**
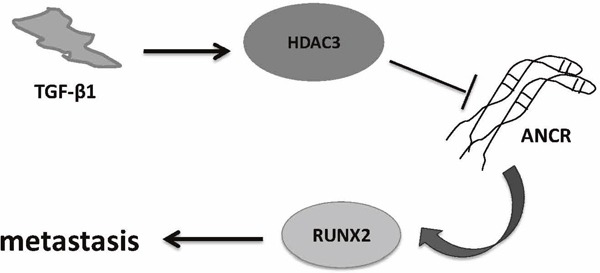
Proposed working model of ‘TGF-β-ANCR-RUNX2 pathway’ in breast cancer metastasis progression TGF-β1 induces HDAC3 increasing; then HDAC3 binding on ANCR promoter region represses ANCR transcription; moreover, ANCR decreasing results in RUNX2 expression increasing and promoting breast cancer metastasis finally.

In conclusion, data presented in this report demonstrates that ANCR is down-regulated by TGF-β signal pathway. Down-regulation of ANCR increases RUNX2 expression and promotes breast cancer cells invasion and metastasis *in vitro* and *in vivo*. As illustrated in the ‘TGF-β-ANCR-RUNX2 pathway’ proposed model (Figure 9). However, due to our limitation in time and funds, further experiments which may contribute to the understanding of the detailed molecular mechanisms that may be involved in the ANCR dependent regulation of TGF-β1 signaling pathway and ANCR regulation of RUNX2 described were not performed. And our collected samples from breast cancer tissues and adjacent tissues is not so many. In the future, these experiments with larger sample size from cancer tissues, adjacent tissues as well as normal tissues will be further conducted to improve our preliminary work and clarify the molecular mechanisms in depth.

Above all, our findings provide a novel insight into the functional role of the ANCR-driven anti-tumorigenesis. This study establishes that ANCR is a tumor suppressor, and ANCR may be a potential diagnostic and therapy biomarker for breast cancer patients.

## MATERIALS AND METHODS

### Specimens and cell lines

All the breast cancer tissues were obtained from the Oncology Hospital of Jilin Province, China. Samples were frozen in liquid nitrogen immediately after surgery. Breast tissue specimens were collected using the protocols approved by the Ethics Committee of the Jilin Oncology Hospital. All the cell lines used in this study were obtained from American Type Culture Collection (ATCC, Manassas, VA, USA). MCF10A cells were cultured in DMEM/F12 (Sigma, St. Louis, MO, USA) with 5% horse serum (Gibco, Grand Island, NY, USA), 20 ng/ml EGF (R&D), 0.5 mg/ml hydrocortisone (Sigma), 100 ng/ml cholera toxin (Sigma), 10 mg/ml insulin (Gibco) and pen/strep. Both MDA-MB-231 cells and MDA-MB-231 high metastasis (MDA-MB-231HM) cells were cultured in Leibovitz’s L-15 medium with 10% FBS at 37^°^Cwithout CO_2_. MCF7, T47D and BT549 cells were cultured in RPMI-1640 (Sigma) medium with 10% FBS (ExCell Bio, Shanghai, China). HEK293T cells were cultured in DMEM (Sigma) with 10% FBS.

### Western blot, plasmids and virus infection

Western Blot, plasmids and virus infection have been shown in our Supplementary Information.

### Antibodies and reagents

The primary antibodies against the following proteins were used: E-cadherin (BD Biosciences 610182, 1:5000), N-cadherin (BD Biosciences 610920, 1:5000), Vimentin (BD Biosciences 550513, 1:8000), Fibronectin (BD Biosciences 610077, 1:2000); β-actin (Sigma-Aldrich A1978, 1:10000); Snail (Abcam ab63371, 1:1000), Slug (Abcam ab27568, 1:1000); Zeb1(Santa Cruz sc-25388, 1:500), HDAC3 (Santa Cruz sc-17795, 1:1000); HDAC1(GeneTex, GTX27028, 1:2000); HDAC2(GeneTex, GTX12169, 1:2000); HDAC4(GeneTex, GTX50484, 1:2000); Smad2/3(Cell Signaling Technology 5678S, 1:1000), p-Smad2/3 (Cell Signaling Technology 8828S, 1:1000), RUNX2(Cell Signaling Technology 12556S, 1:1000); H3-Ac (Millipore #06-599, 1:1000), H4-Ac (Millipore #06-598, 1:1000). The secondary antibodies used were: goat anti-mouse and goat anti-rabbit (ZSGB-BIO, Beijing, China, 1:2000).

Recombinant Human TGF-β1 was purchased from R&D Systems.

### RNA extraction, reverse transcription and real-time-PCR

RNA extraction, reverse transcription and Real-time-PCR experiments were performed basically as described previously [24]. β-actin was used as an internal control. The sequences of PCR primers are listed in Supplementary Materials.

### Wound healing, cell invasion and migration assays

Wound healing, cell invasion and migration assays are shown in our Supplementary Information.

### Luciferase reporter assay

The pGL4-basic dual luciferase vector (Promega) was use to construct ANCR promoter region. ANCR promoter fragment was amplified using the following primers: 5′-GGGGTACCACGGTTTAGGCGGACACA-3′(sense), 5′-CCCAAGCTTCGCGCAACTCCAGCTGAC-3′(antisense). The sequence of the ANCR promoter was validated by direct sequencing. Subsequently, the construct was transiently transfected into HEK293T cells; at the same time Renilla plasmid (Promega) and HDAC3 plasmid were co-transfected. Twenty-four hours later, cells were harvested, and luciferase activities were measured by the Dual-Luciferase Reporter Assay Kit (Promega) on a luminometer (Molecular Devices, Sunnyvale, CA, USA). And ANCR promoter relative firefly luciferase activity was normalized to the Renilla activity.

### Chromatin immunoprecipitation–quantitative PCR

The chromatin immunoprecipitation (ChIP) Kit was purchased from Millipore (Cat No. 17-10085) and ChIP experiments were carried out essentially in accordance with manufacturer’s guidelines. Immnuoprecipitated DNA was amplified with the designated primers on the Roche LightCycler480. The ANCR ChIP-qPCR Primers for ANCR promoter were: 5′- CGCCCTTGCCCAGAGTCTT-3′ (sense) and 5′- GGAGACCGAAAGCCGAAGA-3′ (antisense).

### *In vivo* tumor lung-colonization assays

*In vivo* tumor lung-colonization assays also have shown in the Supplementary Information.

### Statistical analysis

Data are presented as mean ± S.D. The Student *t* -test (2-tailed) was used to determine statistic significance of differences between groups. *P*<0.05 was considered statistically significant. Statistical analysis was performed using the GraphPad Prism software (GraphPad Software, La Jolla, CA, USA). And the Pearson correlation analysis was performed by SPSS 17.0 software.

## SUPPLEMENTARY MATERIALS FIGURES


