# Supplementary material for: LncRNA ANCR down-regulation promotes TGF-β-induced EMT and metastasis in breast cancer
Source: Oncotarget. 2017 Jun 27;8(40):67329–43. doi: 10.18632/oncotarget.18622 (PMC5620176; doi:10.18632/oncotarget.18622)
Supplement: Supplementary file 1 [file oncotarget-08-67329-s001.pdf]

LncRNA ANCR down-regulation promotes TGF-β-induced EMT and metastasis in breast cancer

SUPPLEMENTARY MATERIALS

Sequences of plasmids constructing primers

ANCR expression

S 5'-CGGGATCC CCCGCCCCGCGCCGCCTC TC-3'

AS 5'-CGGAATTC GTCAGGCCAAGTAAGTTT AT-3'

shCtrl

S 5'-GATCCCCTTCTCCGAACGTGTCACGTTT CAAGAGAACGTGACACGTTCGGAGAATTTTTC-3'

AS 5'-TCGAGAAAAATTCTCCGAACGTGTC ACGTTCTCTTGAAACGTGACACGTTCGGAGAAG GG-3'

shANCR#1

S 5'-GATCCCCGCGTACTAACTTGTAGCAATT CAAGAGA TTGCTACAAGTTAGTACGCTTTTTC-3'

AS 5'-TCGAGAAAAAGCGTACTAACTTGTAGC AATCTCTTGAA TTGCTACAAGTTAGTACGCGGG-3'

shANCR#2

S 5'-GATCCCCGAGCTAGAGCAGTGACAATT TCAAGAGAATTGTCACTGCTCTAGCTCTTTTTC-3'

AS 5'-TCGAGAAAAAGAGCTAGAGCAGTGAC AATTCTCTTGAAATTGTCACTGCTCTAGCTCGGG-3'

Sequences of PCR primers (real-time PCR and RT-PCR)

β-actin: S 5'-GAGCACAGAGCCTCGCCTTT-3'
AS 5'-ATCCTTCTGACCCATGCCCA-3'.
ANCR: S 5'- GCCACTATGTAGCGGGTTTC-3'
AS 5'-ACCTGCGCTAAGAACTGAGG-3'
E-cadherin: S 5'-GACAACAAGCCCGAATT-3'
AS 5'-GGAAACTCTCTCGGTCCA-3'.
N-cadherin: S 5'-CGGGTAATCCTCCCAAATCA-3'
AS 5'-CTTTATCCCGGCGTTTCATC-3'.
Vimentin: S 5'-GAGAACTTTGCCGTTGAAGC-3'
AS 5'-GCTTCCTGTAGGTGGCAATC-3'.
Fibronectin: S 5'-CAGTGGGAGACCTCGAGA AG-3'
AS 5'-TCCCTCGGAACATCAGAAAC-3'.
RUNX2: S 5'- CCGCCTCAGTGATTTAGGGC-3'
AS 5'- GGGTCTGTAATCTGACTCTGTCC-3'.

Western blot, plasmids and virus infection

Cells were washed twice in cold PBS and lysed in RIPA lysis buffer (50mM Tris-HCl, pH 7.4, 150mM NaCl, 1% sodium deoxycholate, 1%Triton X-100 and 0.1% SDS) plus protease inhibitor cocktail (Roche, Mannheim, Germany). Protein lysates were subjected to SDS-PAGE, transferred to PVDF membrane (Millipore) and detected with appropriate primary antibodies coupled with HRP-conjugated secondary antibodies by ECL reagent (GE Healthcare, Buckinghamshire, UK).

Plasmids and the human ANCR shRNAs were cloned into pEN_hH1c and then transferred into destination vector pDSL_hpUGIP by LR-clonase (Invitrogen, Camarillo, CA, USA). The full length ANCR and ANCR promoter fragments were obtained by RT-PCR. The shRNA and plasmid cloning primer sequences are listed in Supplementary Information. Lentiviruses were produced by co-transfecting HEK293T cells with one of the expression plasmids and the packaging plasmids (psPAX2 and pMD2.G); the supernatants were collected after 48h, and filtered through 0.45-mm filters (Millipore, Temecula, CA, USA), then concentrated using Amicon Ultra centrifugal filters (Millipore 100KD MWCO). The concentrated viruses were used to infect MCF10A, MCF7 and MDA-MB-231 cells. Stable transfection cell lines were selected with 2 mg/ml puromycin for 15 days. Stable ANCR or shANCR-knockdown cells were generated by lentivirus infection.

Wound healing, cell invasion and migration assays

For wound healing assay, $1x10^6$ cells were plated in 6-well plates. The scratch was done by a 10μl pipette tip. The progression of migration was detected and photographed 24h later. For cell invasion and migration assays, cells were starved for 24 h, and then 5×10^5 (invasion) or 5×10^4 (migration) cells in serum-free media were plated onto the upper chamber with 8 mm pores (Corning, Life Sciences, Lowell, MA, USA), and containing 10% FBS media was placed in the lower chamber. The chambers were coated with fibronectin (FN) (BD Biosciences, San Jose, CA, USA). For invasion assay, the upper chambers were coated with Matrigel

(BD Biosciences) and stained after 48 h. For migration assay, cells were stained with crystal violet (Sigma) after incubation for 16 h. Randomly selected fields were photographed (Nikon ECLIPSE 80i) and stained cells were statistic analyzed.

In vivo tumor lung-colonization assays

For *in vivo* metastasis assay, the BABL/c nude mice were injected with either the MDA-MB-231-Vector or the MDA-MB-231-ANCR cells ($2.5x10^6$) into the tail veins. Lungs were subsequently collected at week 9 and fixed in 10% formalin. The number of metastatic colonies in lungs was counted under a dissection microscope, and the lung tissue sections were prepared and stained with hematoxylin and eosin assays (HE) and immunohistochemistry assays (IHC). For bioluminescence imaging, mice were injected with 150 mg/kg D-luciferin (Goldbio) 10 minutes before imaging. After mice were narcotized, images were collected using the NightOWL LB 983 Spectrum Imaging System. All the animal experiments were approved by the Animal Care Committee of the Northeast Normal University, China. The mice were obtained from the Beijing HFK Bioscience Co., Ltd. (Certification NO. SCXK (Jing) 2009–0004).

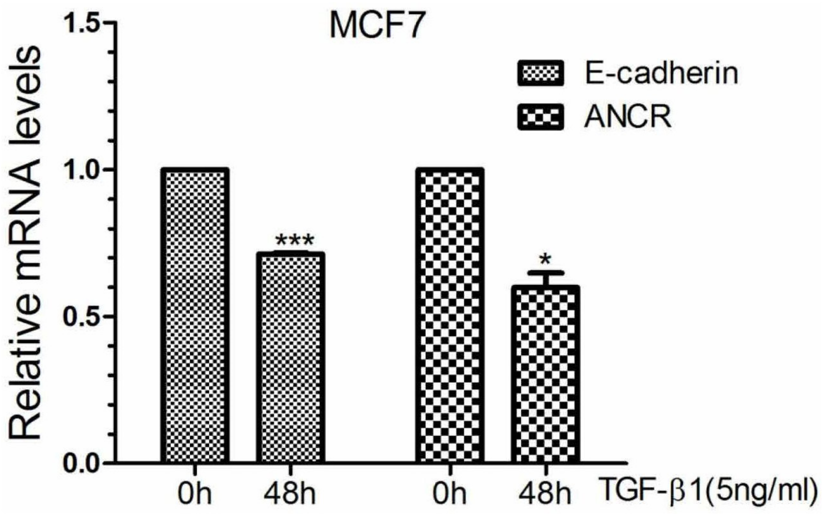

Supplementary Figure 1: ANCR expression is decreased when treated by TGF-β1 in MCF7 cells.

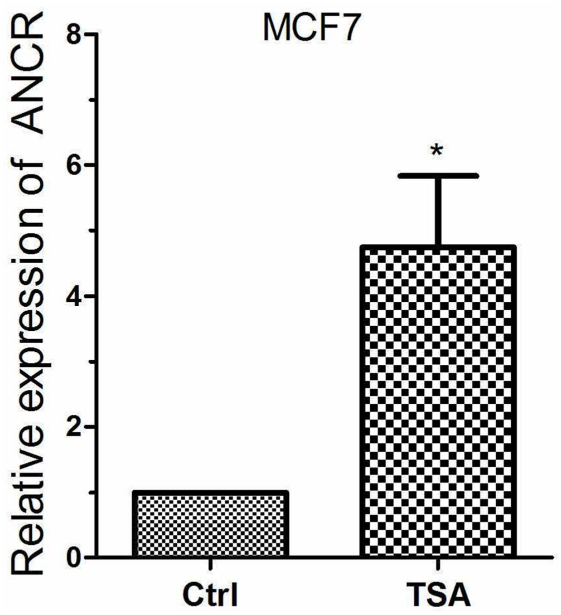

Supplementary Figure 2: ANCR expression is increased when treated by TSA in MCF7 cells.

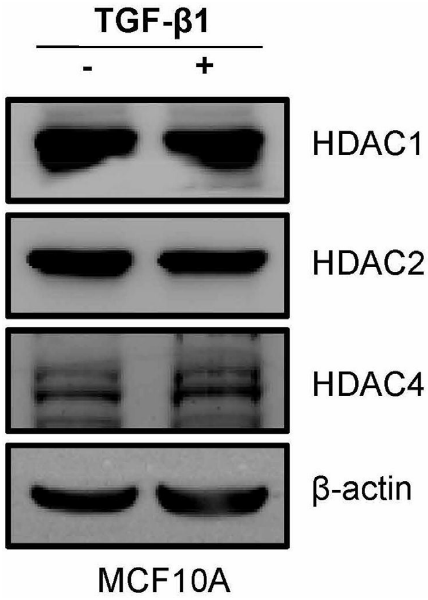

Supplementary Figure 3: Several HDAC family members (HDAC1, HDAC2 and HDAC4) were detected by western blot when treated by TGF-β1 in MCF10A cells.

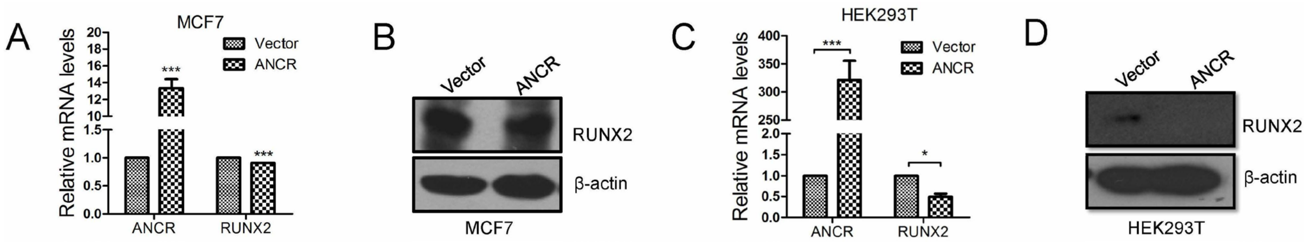

Supplementary Figure 4: RUNX2 expression is repressed by ANCR. (A-B) RUNX2 mRNA and protein expression detected by real-time PCR and western blot assays in MCF7-Vector and MCF7-ANCR cells. **(C-D)** RUNX2 mRNA and protein expression detected in HEK293T-Vector and HEK293T -ANCR cells.
